# Carriage and antimicrobial susceptibility patterns of rectal ESBL *E. coli* in surgical patients at the University Teaching Hospitals in Lusaka, Zambia

**DOI:** 10.1093/jacamr/dlae159

**Published:** 2024-10-09

**Authors:** Amon Siame, Kaunda Yamba, Mulemba Samutela, Andrew Mukubesa, Gina Mulundu

**Affiliations:** Department of Pathology and Microbiology, School of Medicine, University of Zambia, Lusaka, Zambia; Department of Pathology and Microbiology, University Teaching Hospitals, Lusaka, Zambia; Department of Biomedical Sciences, School of Health Sciences, University of Zambia, Lusaka, Zambia; Department of Disease Control, School of Veterinary Medicine, Lusaka, Zambia; Department of Pathology and Microbiology, School of Medicine, University of Zambia, Lusaka, Zambia

## Abstract

**Background:**

Surgical site infections (SSIs) are on the rise and are a global concern as they complicate the recovery of patients postoperatively. Bacterial colonization of the patient’s skin and alimentary tract are known to be major contributing sources to SSIs. However, Zambia lacks data relating to carriage rates of antibiotic-resistant rectal *Escherichia coli* among surgical patients.

**Methods:**

This was a cross-sectional study aimed at determining the preoperative (preop) and postoperative (postop) carriage and antimicrobial susceptibility patterns of rectal ESBL-producing *E. coli* (ESBL-Ec) in elective surgery patients at the highest tertiary hospital in Lusaka, Zambia. Phenotypic methods were used in the identification of *E. coli.* Antibiotic susceptibility patterns and identification of ESBL-Ec was determined by Kirby–Bauer disc diffusion.

**Results:**

A total of 120 study participants were recruited, of which 75 were followed up at least 72 h after surgery. From 195 rectal swabs cultured, 177 (90.8%) were positive for *E. coli*, of which 53 (29.9%) were ESBL-Ec, with a significantly (*P* < 0.0001) higher proportion in postop (47.9%) than preop (17.3%) participants. Overall, ESBL-Ec isolates showed higher resistance in postop than preop to cefotaxime (100% versus 88.9%, respectively), ampicillin (100% versus 94.4%), ciprofloxacin (88.3% versus 83.3%), amoxicillin/clavulanic acid (80% versus 66.7%) and cefepime (80% versus 77.8%). MDR ESBL-Ec strains were more frequent in postop than in preop participants (91.4% versus 88.9%).

**Conclusions:**

The study showed a significantly higher rate of antimicrobial-resistant rectal *E. coli* in postop than preop participants. There is a need to ascertain the source of the resistance and to institute robust infection control measures, preop screening of surgical patients for ESBL-Ec, and to raise awareness on prudent use of antibiotics.

## Introduction

Surgical site infections (SSIs) are a global concern and complicate the recovery course of patients.^1^ SSIs are defined as infections that typically occur within 30 days of an operation at the site or part of the body where the surgery took place, or within a year if an implant is left in place and the infection is thought to be secondary to surgery.^2^ Bacterial colonization of the patient’s skin, gastrointestinal and genital tracts are the principal contributing sources to SSIs. Although Gram-positive bacteria are the main causative organisms in SSIs, Gram-negative bacilli, mostly Enterobacterales such as *Escherichia coli*, are frequently involved. In general, production of ESBLs is one of the most prevalent resistance mechanisms among enteric Gram-negative bacteria (GNB).^3^ ESBL-producing bacteria exhibit effective hydrolysis of β-lactam antibiotics, including broad-spectrum β-lactam drugs and monobactams, except cephamycin and β-lactam inhibitors.^4^ The plasmids bearing genes encoding ESBLs also frequently carry genes that encode resistance to other antimicrobial agents, such as aminoglycosides and quinolones,^5,6^ making these strains highly resistant to a number of commonly prescribed antimicrobial agents.

In Zambia, the burden of resistant strains keeps rising with time and may lead to serious complications in surgical patients.^7,8^ One of the strategies to control resistant strains is screening of patients undergoing elective surgery for possible carriage, including all healthcare staff working in a hospital, to monitor for such resistant strains as a means of infection control.^9^ However, this is not practised at the University Teaching Hospitals (UTH) in Lusaka, Zambia, and as such, this poses serious threats to surgical patients. A Zambian study as recently as 2023 reported that 70.1% of the *E. coli* isolates from clinical specimens at the UTH were ESBL producers.^10^ Another proxy study involving the burden of ESBL-producing *Klebsiella pneumoniae* in a neonatal ICU showed 100% resistance to all antimicrobial agents tested.^11^ Most of the patients admitted to the UTH in Lusaka, Zambia show a relatively high carriage rate of resistant strains of *E. coli*. A study by Nagelkerke and co-workers^8^ reported that 52% of the inpatients were significantly colonized with ESBL-producing *E. coli* (ESBL-Ec) compared with 12% of the outpatients. However, there has been no study conducted in Zambia to assess the carriage of ESBL-Ec in surgical patients. Surgical patients are more at risk of SSIs, the source of which could be due to the surgical procedure or exposure to higher generations of antimicrobial agents such as extended-spectrum β-lactam cephalosporins. Therefore, the current study assessed the preoperative (preop) and postoperative (postop) carriage and antimicrobial susceptibility patterns of rectal ESBL-Ec in patients scheduled for elective surgery at the UTH Adult Hospital in Lusaka, Zambia.

## Materials and methods

This was a cross-sectional study on the carriage and antimicrobial susceptibility patterns of ESBL-Ec isolates from rectal specimens, in surgical patients at the UTH Adult and Emergency Hospital in Lusaka, Zambia. The sampling population comprised all adult male and female patients admitted for less than 48 h at the time of the first sampling prior to surgery. Patients were excluded if they had superficial skin and soft tissue infections, had been hospitalized for more than 48 h at the time of the first sampling, and those who took antibiotics in the last 21 days.

A total of 120 study participants were recruited into the study, from whom 120 rectal swabs were collected prior to surgery, while 75 rectal swabs were collected 72 h after surgery. Systematic random sampling was used during recruitment of the study participants. Participants’ sociodemographic information, antibiotic prescription data and medical history relevant to the study were obtained from the participants’ medical records and through interviews using a structured data collection tool. The study was carried out between September 2021 and April 2022.

Rectal swabs were collected using a sterile cotton-tipped swab in Amies transport medium. The first sampling was done within 48 h of admission and before surgery, to assess carriage status and antibiotic resistance of *E. coli* before hospital stays. This was followed by another rectal swab collected at least 72 h after surgery to assess possible ESBL-Ec carriage status and antibiotic resistance acquired during the hospital stay. Rectal swabs were inoculated primarily on MacConkey (MAC) agar and incubated at 37°C aerobically for 24 h. The plates were then examined for lactose-fermenting colonies suggestive of *E. coli*. Identification of *E. coli* was based on the following characteristics: Gram-negative rods, lactose fermenters on MAC, oxidase negative, citrate negative, indole positive, motility positive, reactions in triple sugar iron agar (TSIA) as acid slant and butt, gas positive and no hydrogen sulphide production, and on lysine iron agar (LIA) as alkaline slant and butt, negative for gas and hydrogen sulphide production. A cocktail of three isolated morphologically similar lactose-fermenting colonies per specimen was suspended in tryptone soy broth, incubated for 4 h, and later subcultured onto new MAC with 2 μg/mL cefotaxime.

The antimicrobial susceptibility pattern of *E. coli* isolates was determined by the standard Kirby–Bauer disc diffusion method and the results were interpreted using the 2021 CLSI M100 guidelines. A suspension of pure colonies (three colonies) of *E. coli* was made in sterile normal saline and equated to 0.5 McFarland standard. A lawn culture was done on Mueller–Hinton agar and the following antibiotic discs were tested: ampicillin (10 µg), cefotaxime (30 µg), ceftriaxone (30 µg), ceftazidime (30 µg), cefepime (30 µg), amoxicillin/clavulanic acid (20/10 µg), piperacillin/tazobactam (100/10 µg), imipenem (10 µg), ciprofloxacin (5 µg), amikacin (30 µg), gentamicin (10 µg), ampicillin/sulbactam (10/10 µg) and cefoxitin (30 µg).

MDR *E. coli* was defined as *E. coli* having acquired non-susceptibility to at least one agent of three or more antimicrobial categories. For quality control of antibiotic discs, *E. coli* ATCC 25922 was used. The antibiotics were selected based on what is commonly used in the treatment of SSIs at the UTH in Lusaka, Zambia. All phenotypically identified *E. coli* (cocktail of three colonies per specimen) were subjected to phenotypic ESBL screening based on *E. coli* strains that were resistant to at least one or more of the three tested third-generation cephalosporins (cefotaxime, ceftriaxone and ceftazidime). Detection of ESBL-Ec was done using two tests in succession. The first test used the double-disc synergy test, which involved placing amoxicillin/clavulanic acid discs (20/10 µg) 20 mm centre-to-centre next to at least three third-generation cephalosporin discs (cefotaxime, ceftriaxone and ceftazidime) to increase the sensitivity of the test.^12^ After 24 h incubation, a positive result was indicated by inhibition zones around third-generation cephalosporins augmented in the direction of amoxicillin/clavulanic acid. The second test for phenotypic confirmation of ESBL-Ec was called the combination disc test.^13,14^ This involved the use of single indicator third-generation cephalosporins ceftazidime and cefotaxime, and cephalosporin/clavulanic acid (ceftazidime/clavulanic acid and cefotaxime/clavulanic acid). These sets of antibiotics were placed on Mueller–Hinton agar plated with a 0.5 McFarland standard suspension of *E. coli*. The plates were then incubated aerobically at 37°C for 24 h. Presumptive ESBL production was indicated by the difference in the zone size of inhibition between cephalosporin/clavulanic acid and single indicator cephalosporins being ≥5 mm.

## Data analysis

Raw data were entered into an Excel spreadsheet, audited, cleaned and coded and then exported to SPSS version 28 (IBM SPSS Statistics for Windows, Armonk, NY, USA) for analysis. Descriptive statistics were computed for all variables as frequencies and proportions, and the average for age was computed as median and IQR (because age was not normally distributed by the Shapiro–Wilk test; *P* < 0.05). The chi-squared test was done to determine the relationship between the carriage rate of rectal ESBL-Ec and surgical status (preop versus postop); Fisher’s exact test was done for cells with fewer than five counts. Tests were interpreted at 5% significance level (two-sided) and 95% CI, and a *P* value of <0.05 was considered statistically significant.

### Ethics

This study was approved by the University of Zambia Biomedical and Research Ethics Committee (UNZABREC; REF 923-2020) and the National Health Research Authority (NHRA). Signed informed consent was obtained from eligible study participants prior to enrolment. Access to patient-related data was restricted to study personnel, and all patient identifiers were kept confidential. To ensure confidentiality and anonymity, the participants’ samples and isolates were assigned project identification numbers.

## Results

A total of 120 study participants were recruited prior to surgery. Most of the study participants were from urology (82; 72.6%), while the rest came from general surgery and orthopaedics. Extended-spectrum cephalosporins (cefotaxime and ceftriaxone) were the most frequently prescribed antibiotics both preop and postop, with a higher proportion of cefotaxime than ceftriaxone, as shown in Table 1. A total of 195 rectal swabs were cultured, with 120 and 75 from preop and postop patients, respectively. *E. coli* was detected in 104 out of 120 preop rectal swabs and 73 were positive for *E. coli* from 75 postop rectal swabs. The number with carriage of ESBL-Ec was 53 (29.9%), with a statistically significant (*P* < 0.0001) higher proportion in postop (47.9%) than preop (17.3%) participants. Regarding carriage of ESBL-Ec, 18 patients had ESBL-Ec both preop and postop, while for 27 who initially had no ESBL-Ec preop, their samples contained the ESBL-Ec postop. ESBL-Ec carriage was proportionately higher among male than female patients, and significantly (*P* < 0.05) higher among urology and orthopaedic patients postop than patients from general surgery, as shown in Table 2.

**Table 1.** Preop and postop antibiotic prescriptions by surgical category

Variables	Surgery category			Total
	General surgery (*N* = 12)	Orthopaedic (*N* = 19)	Urology (*N* = 82)	
Preop antibiotics given, *n* (%)				
Ceftriaxone	3 (25)	1 (5.3)	17 (20.7)	21 (18.6)
Cefotaxime	0 (0)	5 (26.3)	32 (39)	37 (32.7)
None	9 (75)	13 (68.4)	33 (40.2)	55 (48.7)
Postop antibiotics given, *n* (%)				
Ciprofloxacin	0 (0)	0 (0)	3 (3.7)	3 (2.7)
Ceftriaxone	3 (25)	5 (26.3)	21 (25.6)	29 (25.7)
Cefotaxime	1 (8.3)	6 (31.6)	31 (37.8)	38 (33.6)
Cefotaxime and metronidazole	2 (16.7)	2 (10.5)	1 (1.2)	5 (4.4)
Metronidazole only	1 (8.3)	0 (0)	0 (0)	1 (0.9)
Nitrofurantoin	0 (0)	0 (0)	2 (2.4)	2 (1.8)
None	5 (41.7)	6 (31.6)	24 (29.3)	35 (31)

**Table 2.** Preop and postop isolation rates of rectal *E. coli* and ESBL-Ec

Variable description	Preop phase	Postop phase	*P* value	Total
Samples cultured	120	75		195
*E. coli* isolation, *n* (%)				
Positive	104 (86.7)	73 (97.3)	0.0152	177 (90.8)
Negative	16 (13.3)	2 (2.7)		18 (9.2)
ESBL-Ec carriage, *n* (%)				
Positive	18/104 (17.3)	35/73 (47.9)	*P *< 0.0001	53 (29.9)
Negative	86/104 (82.7)	38/73 (52.1)		124 (70.1)

### Antimicrobial resistance pattern of rectal E. coli

Generally, the antibiotic resistance rate was significantly (*P* < 0.05) higher in the postop phase than in the preop phase. Except for ampicillin (preop = 86.3% and postop = 77.9%; *P* = 0.159), piperacillin/tazobactam (preop = 1.9% and postop = 4.1%; *P* = 0.384) and amikacin (preop = 2.9% and postop = 5.5%; *P* = 0.384), to which resistance was not significantly different between preop and postop, most of the antibiotics showed a significantly (*P* < 0.05) higher rate of resistance postop than preop. Further, proportions of MDR *E. coli* isolates were significantly higher postop (33; 45.2%) compared with preop (17; 16.3%), as shown in Table 3.

**Table 3.** Antibiotic resistance pattern of *E. coli* (*n* = 177) isolated from rectal swabs

Antibiotics	Preop (*N*= 104)	Postop (*N* = 73)	*P* value
	*n* (%R)	*n* (%R)	
Penicillins			
Ampicillin	81 (77.9)	63 (86.3)	0.159
Ampicillin/sulbactam	6 (5.8)	12 (16.4)	0.022
Amoxicillin/clavulanic acid	22 (21.2)	35 (47.9)	0.0002
Piperacillin/tazobactam	2 (1.9)	3 (4.1)	0.384^a^
Cephems			
Cefotaxime	15 (14.4)	35 (47.9)	<0.0001
Ceftriaxone	14 (13.5)	34 (46.6)	<0.0001
Ceftazidime	13 (12.5)	35 (47.9)	<0.0001
Cefepime	12 (11.5)	30 (41.1)	<0.0001
Cefoxitin	12 (11.5)	17 (23.6)	0.033
Aminoglycosides			
Amikacin	3 (2.9)	4 (5.5)	0.384^a^
Gentamicin	11 (10.8)	21 (28.8)	0.002
Carbapenem			
Imipenem	0 (0)	0 (0)	
Quinolone			
Ciprofloxacin	29 (27.9)	39 (53.4)	0.0006
MDR strains	17 (16.3)	33 (45.2)	<0.0001
ESBL-Ec	18 (17.3)	35 (47.9)	<0.0001

%R, percentage resistant.

^a^Fisher’s exact test.

Among the penicillin antibiotics, the proportion with resistance to ampicillin (preop = 77.9% and postop = 86.3%) and amoxicillin/clavulanic acid (preop = 21.2% and postop = 47.9%) was higher in the postop phase than in the preop phase. The proportions with resistance to third- and fourth-generation cephalosporins, gentamicin and ciprofloxacin were equally higher in the postop phase than in the preop phase. The proportion with resistance to cefotaxime, compared with other cephalosporins, was significantly (*P* < 0.05) higher postop than preop (47.9% versus 14.4%). The proportion of MDR ESBL-Ec among the 53 isolates was 90.6%, with 88.9% and 91.4% in the preop and postop phase, respectively. Urology and orthopaedics patients showed a significantly higher proportion of ESBL-Ec in the postop phase than in the preop phase (Table 4).

**Table 4.** Antibiotic resistance patterns and sociodemographic characteristics of participants with ESBL-Ec

Variables	Surgical phase		Total	*P* value
	Preop (*N*= 18)	Postop (*N* = 35)		
	*n* (%)	*n* (%)		
Age (years); median (IQR) = 42 (27)				
18–27	1 (5.6)	3 (8.6)	4	0.457
28–37	2 (11.1)	6 (17.1)	8	0.262
38–47	7 (38.9)	19 (54.3)	26	0.711
48–57	2 (11.1)	1 (2.9)	3	0.396
≥58	6 (33.3)	6 (17.1)	12	0.560
Gender				
Male	13 (72.2)	19 (54.3)	32	0.211
Female	5 (27.8)	16 (45.7)	21	0.211
Surgical category				
General surgery	1 (5.6)	6 (17.1)	7	0.113
Orthopaedics	1 (5.6)	8 (22.9)	9	0.039
Urology	16 (88.6)	21 (60.0)	37	0.003
Penicillins				
Ampicillin	17 (94.4)	35 (100)	52	0.162
Ampicillin/sulbactam	6 (33.3)	8 (22.9)	14	0.421
Amoxicillin/clavulanic acid	12 (66.7)	28 (80)	42	0.291
Piperacillin/tazobactam	4 (22.2)	1 (2.9)	5	0.024
Cephems/cephamycins				
Cefotaxime	16 (88.9)	35 (100)	53	0.047
Ceftriaxone	15 (83.3)	34 (97.1)	49	0.075
Ceftazidime	15 (83.3)	34 (97.1)	49	0.075
Cefepime	14 (77.8)	28 (80)	42	0.853
Cefoxitin	11 (61.1)	15 (42.9)	26	0.214
Aminoglycosides				
Amikacin	2 (11.1)	2 (5.7)	4	0.485
Gentamicin	7 (38.9)	18 (51.4)	25	0.393
Quinolone				
Ciprofloxacin	15 (83.3)	31 (88.6)	46	0.593
MDR strains	16 (88.9)	32 (91.4)	48	0.770

## Discussion

This study investigated the carriage and antimicrobial susceptibility patterns of rectal ESBL-producing *E. coli* among 120 recruited surgical patients admitted to the highest tertiary adult hospital in Lusaka, Zambia. Screening for carriage of these resistant bacteria is important in infection prevention, especially among patients undergoing surgery, due to their aetio-pathological association with SSIs. In addition, the alimentary tract is a common site for ESBL-Ec, where they reside as resistant bacterial flora.

The carriage of ESBL-Ec in this study was 29.9%, with more resistant strains detected in the postop phase than in the preop phase (47.9% versus 17.3%). The carriage of ESBL-Ec preop is similar to that in a Mexican study that reported 17.5%, which increased to 21% following a hospital stay.^15^ This showed that the longer the surgical patient stays in the hospital the higher the risk of carriage of ESBL-Ec. Similarly, a study in Israel reported a lower carriage of ESBL-Ec, at 13.8% following colorectal surgery.^16^ The proportion of ESBL-Ec in the preop patients in the current study is very similar to the global rise of resistant *E. coli* strains in the general population, especially in developing countries.^17–19^ The possible explanation for this high proportion of ESBL-Ec in the current study could be due to increased and perhaps uncontrolled exposure to third-generation cephalosporins prior to surgery, resulting in acquisition or development of antibiotic resistance genes. These findings clearly imply there is a risk to patients undergoing elective surgery, particularly surgery involving the abdomen, intestine and the urinary tract. Furthermore, the finding is strongly suggestive of possible occurrence of hospital-acquired infections or colonization, as an indication of poor infection control and prevention measures, as well as the lack of monitoring of antimicrobial use by the antimicrobial stewardship committee.

Patients with urological conditions had a significantly higher proportion of ESBL-Ec after surgery than in the preop phase. This finding could be attributed to the proximity of the genitourinary tract and the alimentary tract (where the *E. coli* is resident), as well as mechanical bacterial transfer between these two anatomical sites during surgery. Other factors attributed to increased colonization of ESBL-Ec among urological patients after surgery may include antibiotic use, poor hygiene, contamination from the environment and acquisition of resistance genes.^20^ Other studies have reported that underlying poor health, bowel surgery, indwelling catheterization and dialysis are other factors that can increase the risk of acquiring healthcare-associated resistant ESBL-Ec.^1,20–22^ The increase in resistance to third-generation cephalosporins in the current study could be attributed to possible overuse and maybe even misuse of cefotaxime or ceftriaxone prior to surgery. The effect of antibiotic overuse among surgical patients could also create an artificial direct or indirect selective pressure resulting in an environmental landscape that allows organisms with novel mutations or newly acquired characteristics to survive and proliferate, especially among hospitalized and chronically ill patients.^23,24^ The use of antibiotics generally alters the composition of the gut flora, thereby leading to the selection of highly resistant bacteria that may translocate through the gut epithelium into the bloodstream and induce healthcare-associated infections. The rise in antibiotic resistance among post-surgical patients further narrows the choice of antibiotic therapy, complicates wound management and prolongs hospital stay.

High rates of resistance to ampicillin, ciprofloxacin and gentamicin observed in the current study mimic findings of another Zambian study done in the Eastern Province.^8^ This high resistance rate to ciprofloxacin has also been demonstrated in sub-Saharan Africa, and the reason could be attributed to its increased use in other infections such as urinary tract infection, other urogenital infections, sepsis and intestinal infections, including over-the-counter purchase without proper diagnosis and prescription.^25,26^ Amikacin and other antibiotics such as piperacillin/tazobactam and ampicillin/sulbactam could be possible alternatives for treating SSIs in our setting, as indicated by low resistance to them, a finding that could be attributed to less misuse due to the complex mode of administering, scarcity, cost and reserving of amikacin for MDR TB treatment.^27^ This high rate of susceptibility to imipenem in all ESBL-Ec, in both preop and postop phases, provides more treatment options for surgical patients who develop SSIs. Though costly, carbapenems serve as last-resort antibiotics for life-threatening infections caused by ESBL-producing strains. They are also a last-resort treatment for MDR infections, as reported in different studies including from France, Sri Lanka, Uganda and Zambia.^26,28,29^ However, due to increased trends of antimicrobial resistance, it is important to restrict the use of carbapenems to only when necessary and with guidance from the antimicrobial stewardship committee, coupled with the regularly updated hospital antibiogram.

The current study also showed high numbers of MDR strains of *E. coli.* This finding is consistent with another study conducted in the Eastern part of Zambia in which inpatients were highly colonized by MDR strains of *E. coli* and *K. pneumoniae.*^8^ The high numbers of MDR ESBL-Ec could be attributed to multiple resistance genes located on the same plasmid, coupled with multiple mutations.^19^ The recent rise in rectal MDR ESBL-Ec could limit the everyday choice of antibiotic treatment, thereby complicating the clinical course of infections, prolonging hospital stay, raising healthcare costs and increasing morbidity and mortality.^30^

The results of this study clearly indicate the presence of ESBL-Ec within the hospital environment. Therefore, decolonization, reducing length of hospital stays, improving hygiene and upscaling other standard infection prevention practices within the hospital environment is key to the reduction in transmission and colonization by these resistant pathogens among surgical patients. Overall, an active hospital antimicrobial stewardship committee could help promote the prudent use of antimicrobial medicines, thereby reducing the burden of antimicrobial resistance in the hospital and the community.

### Study limitations

The sampling method used was systematic random sampling, which could have introduced selection bias. Findings of this study are limited to the surgical wards at the UTH Adult Hospital in Lusaka and cannot be generalized for Zambia. Furthermore, this study had a cross-sectional design, therefore the findings of this study are not causal but build hypothetical arguments regarding the carriage of ESBL-Ec in surgical patients. The study did not evaluate risk factors for the carriage of ESBL-Ec but made a reference to some variables that showed a potential relationship with the outcome. Another potential limitation is that the current study did not include any lactose non-fermenting *E. coli* in the screening for ESBL-Ec in rectal specimens. There were more patients who were recruited in the preop phase than those who were followed up postop. This was because the study was conducted at the peak of the COVID-19 pandemic and most patients were discharged within 72 h. Therefore, the findings of this study must be interpretated with caution.

### Conclusions

The carriage rate of rectal ESBL-Ec was higher in the postop phase than in the preop phase. Most ESBL-Ec isolates were MDR but demonstrated less resistance to amikacin and β-lactam/β-lactam inhibitor combinations (piperacillin/tazobactam, amoxicillin/clavulanic acid and ampicillin/sulbactam). There was no resistance to imipenem recorded. The high carriage rate of resistant strains of rectal ESBL-Ec among surgical patients, especially after surgery, is worrisome and warrants a deliberate policy to screen and decolonize patients prior to surgery. A well-functioning microbiology laboratory and antimicrobial stewardship programme are essential for guidance on evidence-based antibiotic treatment guidelines and continuous sensitization on prudent antibiotic use.
